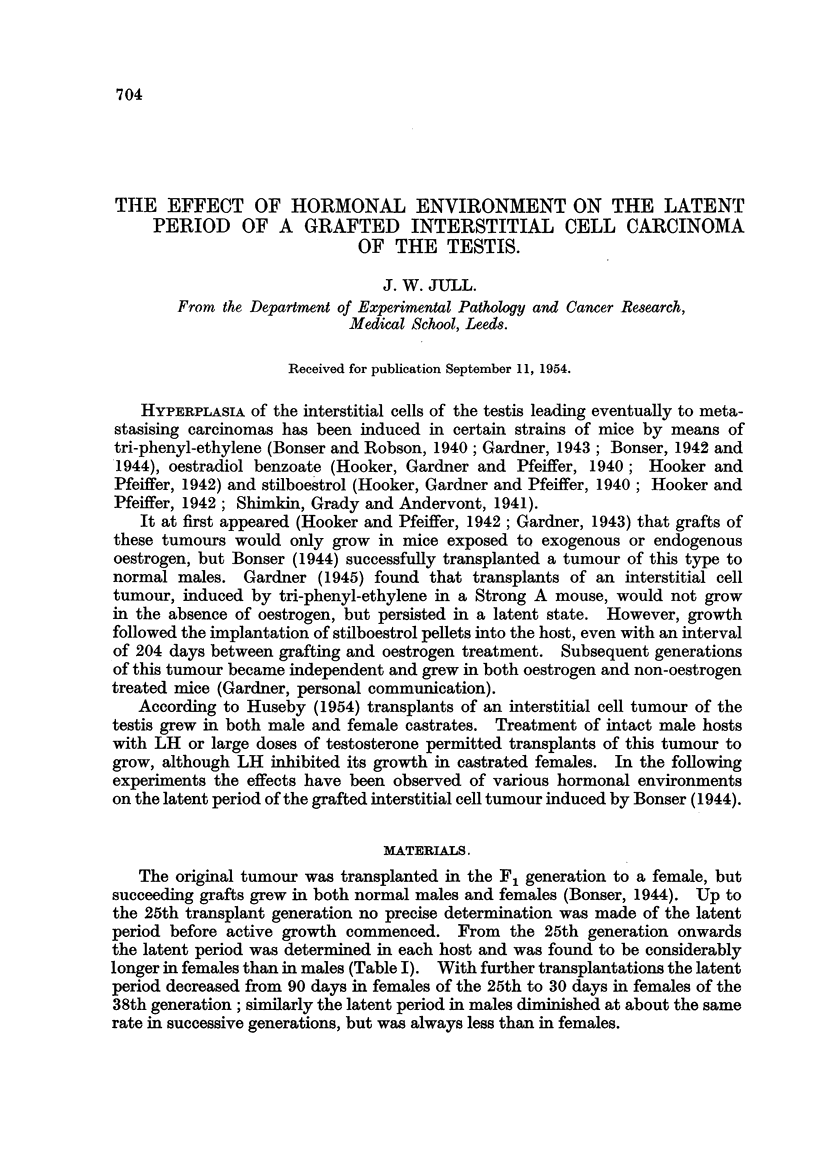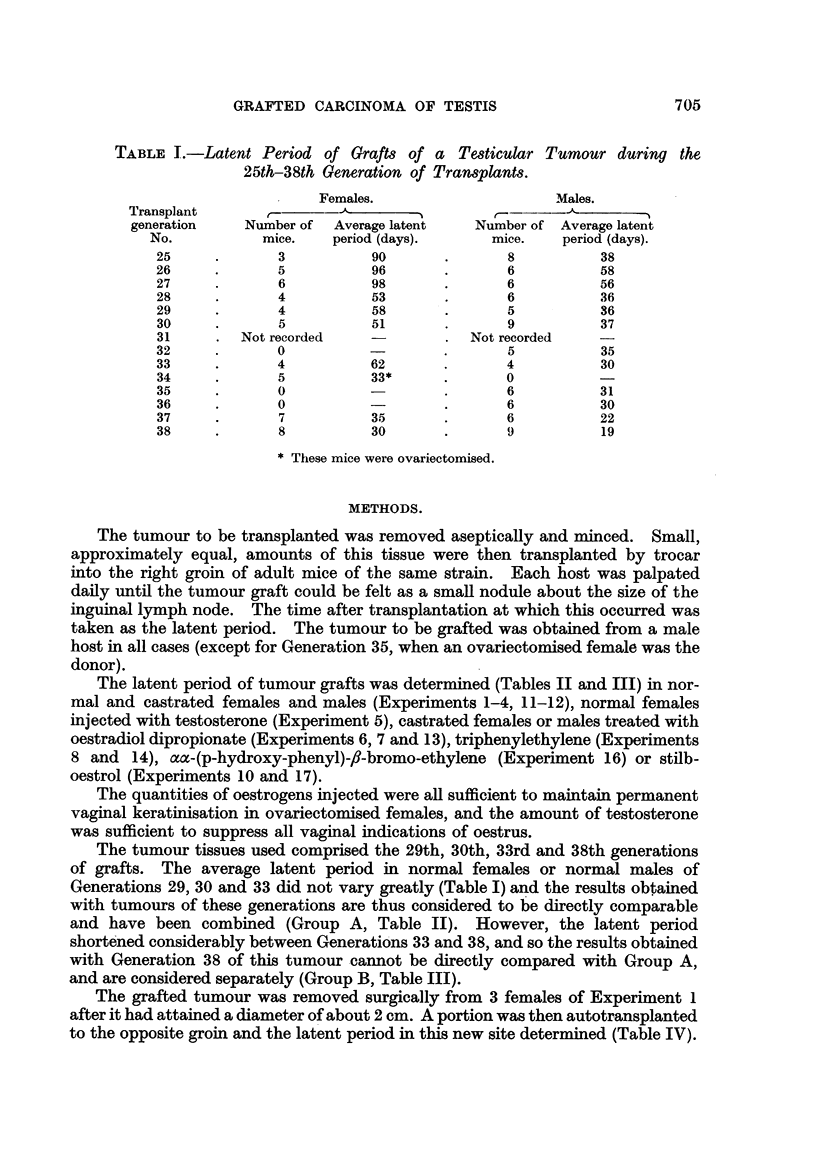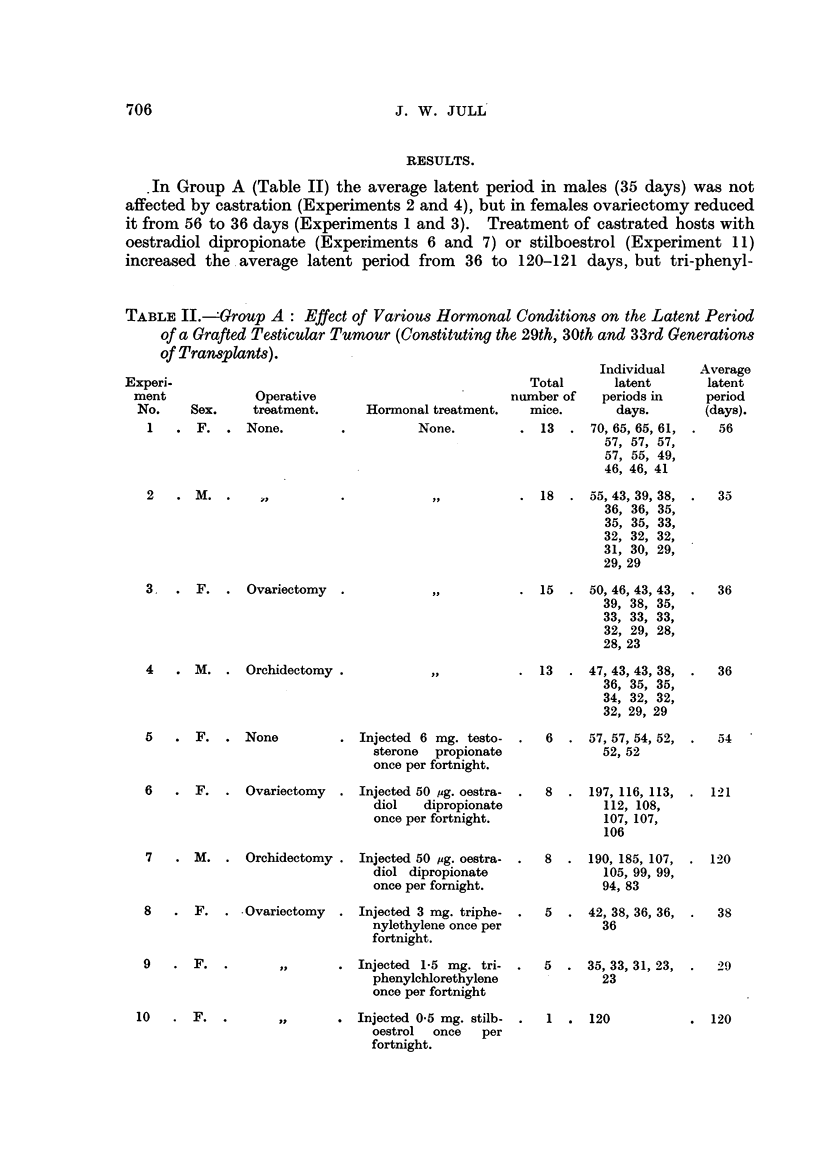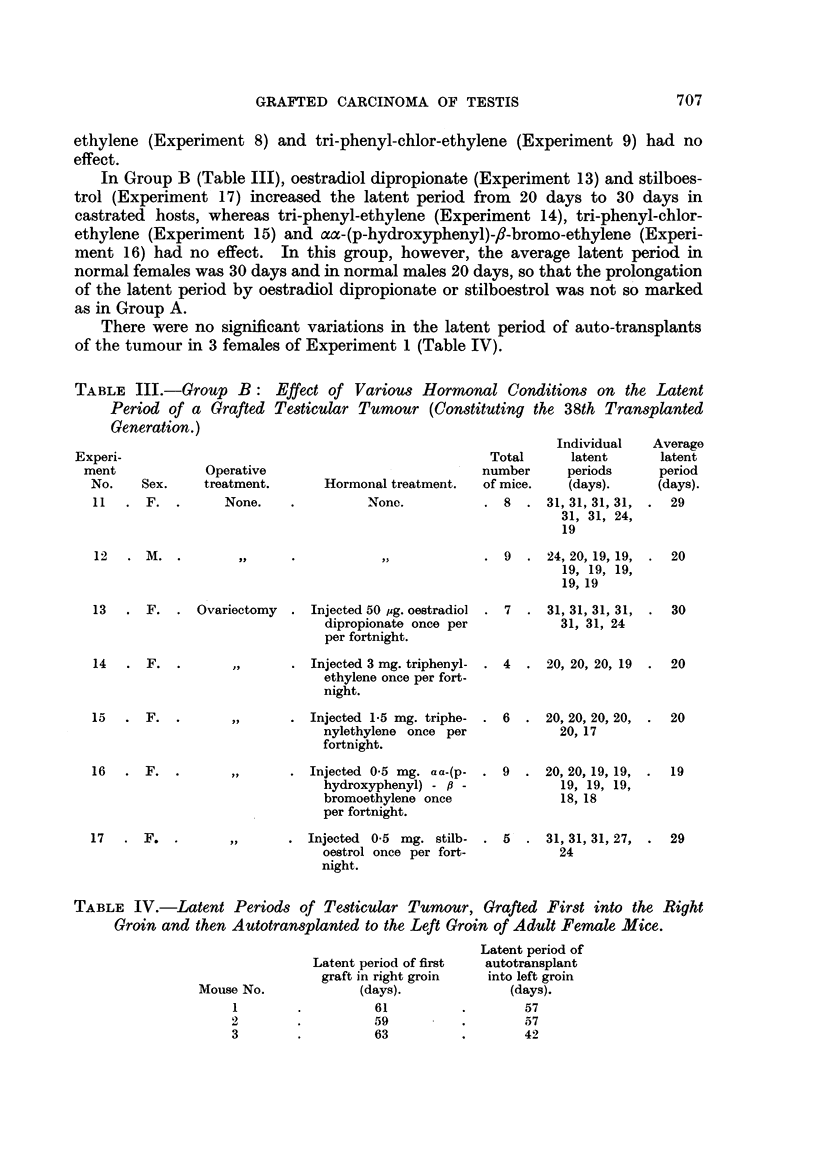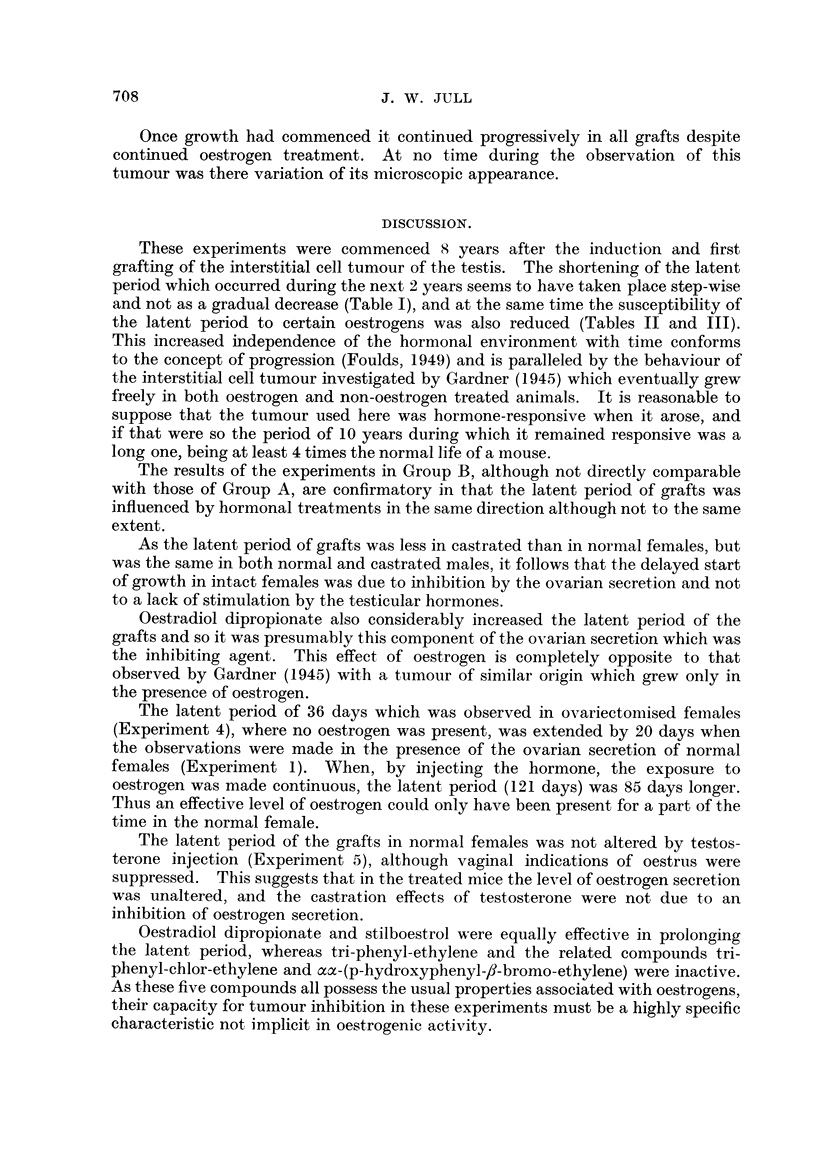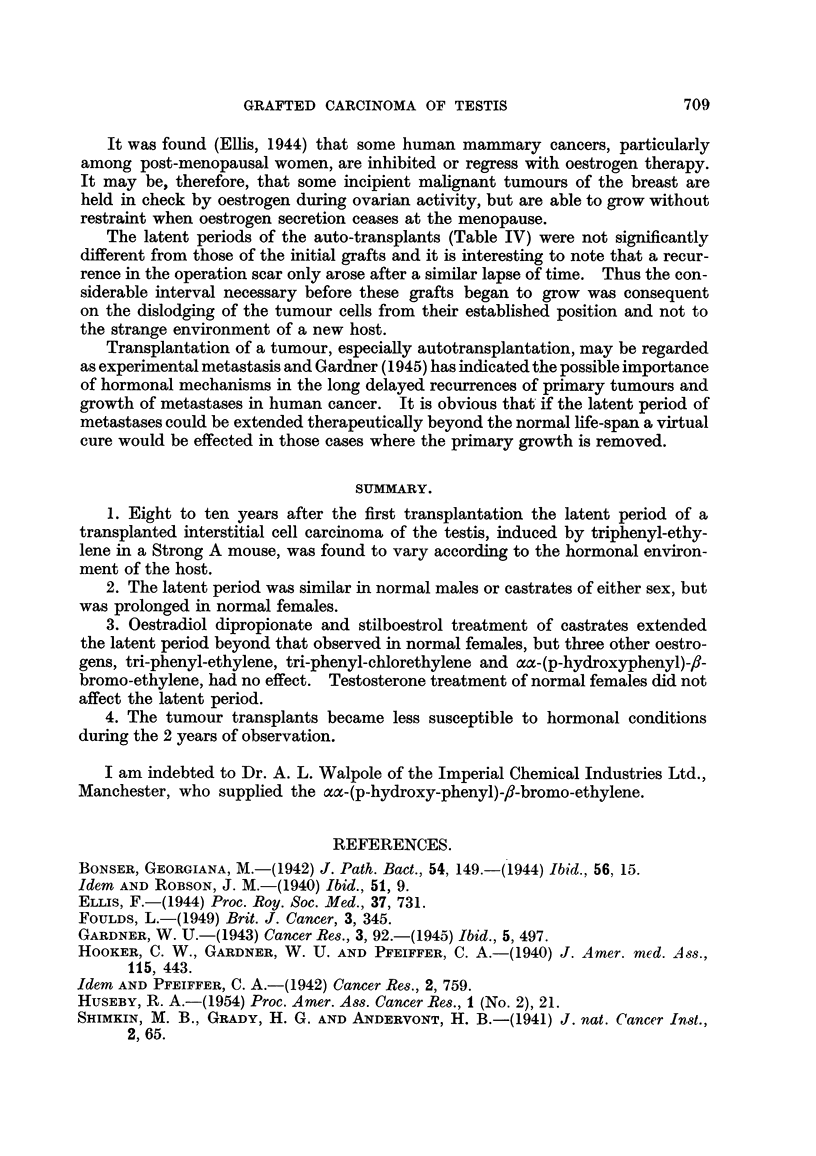# The Effect of Hormonal Environment on the Latent Period of a Grafted Interstitial Cell Carcinoma of the Testis

**DOI:** 10.1038/bjc.1954.77

**Published:** 1954-12

**Authors:** J. W. Jull


### THE EFFECT OF HORMONAL ENVIRONMENT ON THE LATENT PERIOD OF A GRAFTED INTERSTITIAL CELL CARCINOMA OF THE TESTIS.

J. W. JULL.

*From the Department of Experimental Pathology and Cancer Research, Medical School, Leeds.*

Received for publication September 11, 1954.

HYPERPLASIA of the interstitial cells of the testis leading eventually to metastasising carcinomas has been induced in certain strains of mice by means of tri-phenyl-ethylene (Bonser and Robson, 1940 ; Gardner, 1943 ; Bonser, 1942 and 1944), oestradiol benzoate (Hooker, Gardner and Pfeiffer, 1940 ; Hooker and Pfeiffer, 1942) and stilboestrol (Hooker, Gardner and Pfeiffer, 1940 ; Hooker and Pfeiffer, 1942 ; Shimkin, Grady and Andervont, 1941).

It at first appeared (Hooker and Pfeiffer, 1942 ; Gardner, 1943) that grafts of these tumours would only grow in mice exposed to exogenous or endogenous oestrogen, but Bonser (1944) successfully transplanted a tumour of this type to normal males. Gardner (1945) found that transplants of an interstitial cell tumour, induced by tri-phenyl-ethylene in a Strong A mouse, would not grow in the absence of oestrogen, but persisted in a latent state. However, growth followed the implantation of stilboestrol pellets into the host, even with an interval of 204 days between grafting and oestrogen treatment. Subsequent generations of this tumour became independent and grew in both oestrogen and non-oestrogen treated mice (Gardner, personal communication).

According to Huseby (1954) transplants of an interstitial cell tumour of the testis grew in both male and female castrates. Treatment of intact male hosts with LH or large doses of testosterone permitted transplants of this tumour to grow, although LH inhibited its growth in castrated females. In the following experiments the effects have been observed of various hormonal environments on the latent period of the grafted interstitial cell tumour induced by Bonser (1944).

### MATERIALS.

The original tumour was transplanted in the $F_1$ generation to a female, but succeeding grafts grew in both normal males and females (Bonser, 1944). Up to the 25th transplant generation no precise determination was made of the latent period before active growth commenced. From the 25th generation onwards the latent period was determined in each host and was found to be considerably longer in females than in males (Table I). With further transplantations the latent period decreased from 90 days in females of the 25th to 30 days in females of the 38th generation ; similarly the latent period in males diminished at about the same rate in successive generations, but was always less than in females.

TABLE I.—*Latent Period of Grafts of a Testicular Tumour during the 25th–38th Generation of Transplants.*

| Transplant generation No. | Females. Number of mice. | Females. Average latent period (days). | Males. Number of mice. | Males. Average latent period (days). |
|---|---|---|---|---|
| 25 | 3 | 90 | 8 | 38 |
| 26 | 5 | 96 | 6 | 58 |
| 27 | 6 | 98 | 6 | 56 |
| 28 | 4 | 53 | 6 | 36 |
| 29 | 4 | 58 | 5 | 36 |
| 30 | 5 | 51 | 9 | 37 |
| 31 | Not recorded | — | Not recorded | — |
| 32 | 0 | — | 5 | 35 |
| 33 | 4 | 62 | 4 | 30 |
| 34 | 5 | 33* | 0 | — |
| 35 | 0 | — | 6 | 31 |
| 36 | 0 | — | 6 | 30 |
| 37 | 7 | 35 | 6 | 22 |
| 38 | 8 | 30 | 9 | 19 |

* These mice were ovariectomised.

### METHODS.

The tumour to be transplanted was removed aseptically and minced. Small, approximately equal, amounts of this tissue were then transplanted by trocar into the right groin of adult mice of the same strain. Each host was palpated daily until the tumour graft could be felt as a small nodule about the size of the inguinal lymph node. The time after transplantation at which this occurred was taken as the latent period. The tumour to be grafted was obtained from a male host in all cases (except for Generation 35, when an ovariectomised female was the donor).

The latent period of tumour grafts was determined (Tables II and III) in normal and castrated females and males (Experiments 1–4, 11–12), normal females injected with testosterone (Experiment 5), castrated females or males treated with oestradiol dipropionate (Experiments 6, 7 and 13), triphenylethylene (Experiments 8 and 14), $\alpha\alpha$-(p-hydroxy-phenyl)-$\beta$-bromo-ethylene (Experiment 16) or stilboestrol (Experiments 10 and 17).

The quantities of oestrogens injected were all sufficient to maintain permanent vaginal keratinisation in ovariectomised females, and the amount of testosterone was sufficient to suppress all vaginal indications of oestrus.

The tumour tissues used comprised the 29th, 30th, 33rd and 38th generations of grafts. The average latent period in normal females or normal males of Generations 29, 30 and 33 did not vary greatly (Table I) and the results obtained with tumours of these generations are thus considered to be directly comparable and have been combined (Group A, Table II). However, the latent period shortened considerably between Generations 33 and 38, and so the results obtained with Generation 38 of this tumour cannot be directly compared with Group A, and are considered separately (Group B, Table III).

The grafted tumour was removed surgically from 3 females of Experiment 1 after it had attained a diameter of about 2 cm. A portion was then autotransplanted to the opposite groin and the latent period in this new site determined (Table IV).

### RESULTS.

In Group A (Table II) the average latent period in males (35 days) was not affected by castration (Experiments 2 and 4), but in females ovariectomy reduced it from 56 to 36 days (Experiments 1 and 3). Treatment of castrated hosts with oestradiol dipropionate (Experiments 6 and 7) or stilboestrol (Experiment 11) increased the average latent period from 36 to 120–121 days, but tri-phenyl-

TABLE II.—*Group A : Effect of Various Hormonal Conditions on the Latent Period of a Grafted Testicular Tumour (Constituting the 29th, 30th and 33rd Generations of Transplants).*

| Experiment No. | Sex. | Operative treatment. | Hormonal treatment. | Total number of mice. | Individual latent periods in days. | Average latent period (days). |
|---|---|---|---|---|---|---|
| 1 | F. | None. | None. | 13 | 70, 65, 65, 61, 57, 57, 57, 57, 55, 49, 46, 46, 41 | 56 |
| 2 | M. | „ | „ | 18 | 55, 43, 39, 38, 36, 36, 35, 35, 35, 33, 32, 32, 32, 31, 30, 29, 29, 29 | 35 |
| 3 | F. | Ovariectomy | „ | 15 | 50, 46, 43, 43, 39, 38, 35, 33, 33, 33, 32, 29, 28, 28, 23 | 36 |
| 4 | M. | Orchidectomy | „ | 13 | 47, 43, 43, 38, 36, 35, 35, 34, 32, 32, 32, 29, 29 | 36 |
| 5 | F. | None | Injected 6 mg. testosterone propionate once per fortnight. | 6 | 57, 57, 54, 52, 52, 52 | 54 |
| 6 | F. | Ovariectomy | Injected 50 μg. oestradiol dipropionate once per fortnight. | 8 | 197, 116, 113, 112, 108, 107, 107, 106 | 121 |
| 7 | M. | Orchidectomy | Injected 50 μg. oestradiol dipropionate once per fornight. | 8 | 190, 185, 107, 105, 99, 99, 94, 83 | 120 |
| 8 | F. | Ovariectomy | Injected 3 mg. triphenylethylene once per fortnight. | 5 | 42, 38, 36, 36, 36 | 38 |
| 9 | F. | „ | Injected 1·5 mg. triphenylchlorethylene once per fortnight | 5 | 35, 33, 31, 23, 23 | 29 |
| 10 | F. | „ | Injected 0·5 mg. stilboestrol once per fortnight. | 1 | 120 | 120 |

ethylene (Experiment 8) and tri-phenyl-chlor-ethylene (Experiment 9) had no effect.

In Group B (Table III), oestradiol dipropionate (Experiment 13) and stilboestrol (Experiment 17) increased the latent period from 20 days to 30 days in castrated hosts, whereas tri-phenyl-ethylene (Experiment 14), tri-phenyl-chlor-ethylene (Experiment 15) and $\alpha\alpha$-(p-hydroxyphenyl)-$\beta$-bromo-ethylene (Experiment 16) had no effect. In this group, however, the average latent period in normal females was 30 days and in normal males 20 days, so that the prolongation of the latent period by oestradiol dipropionate or stilboestrol was not so marked as in Group A.

There were no significant variations in the latent period of auto-transplants of the tumour in 3 females of Experiment 1 (Table IV).

TABLE III.—*Group B: Effect of Various Hormonal Conditions on the Latent Period of a Grafted Testicular Tumour (Constituting the 38th Transplanted Generation.)*

| Experiment No. | Sex. | Operative treatment. | Hormonal treatment. | Total number of mice. | Individual latent periods (days). | Average latent period (days). |
|---|---|---|---|---|---|---|
| 11 | F. | None. | None. | 8 | 31, 31, 31, 31, 31, 31, 24, 19 | 29 |
| 12 | M. | ,, | ,, | 9 | 24, 20, 19, 19, 19, 19, 19, 19, 19 | 20 |
| 13 | F. | Ovariectomy | Injected 50 μg. oestradiol dipropionate once per per fortnight. | 7 | 31, 31, 31, 31, 31, 31, 24 | 30 |
| 14 | F. | ,, | Injected 3 mg. triphenylethylene once per fortnight. | 4 | 20, 20, 20, 19 | 20 |
| 15 | F. | ,, | Injected 1·5 mg. triphenylethylene once per fortnight. | 6 | 20, 20, 20, 20, 20, 17 | 20 |
| 16 | F. | ,, | Injected 0·5 mg. $\alpha\alpha$-(p-hydroxyphenyl) - $\beta$ - bromoethylene once per fortnight. | 9 | 20, 20, 19, 19, 19, 19, 19, 18, 18 | 19 |
| 17 | F. | ,, | Injected 0·5 mg. stilboestrol once per fortnight. | 5 | 31, 31, 31, 27, 24 | 29 |

TABLE IV.—*Latent Periods of Testicular Tumour, Grafted First into the Right Groin and then Autotransplanted to the Left Groin of Adult Female Mice.*

| Mouse No. | Latent period of first graft in right groin (days). | Latent period of autotransplant into left groin (days). |
|---|---|---|
| 1 | 61 | 57 |
| 2 | 59 | 57 |
| 3 | 63 | 42 |

Once growth had commenced it continued progressively in all grafts despite continued oestrogen treatment. At no time during the observation of this tumour was there variation of its microscopic appearance.

### DISCUSSION.

These experiments were commenced 8 years after the induction and first grafting of the interstitial cell tumour of the testis. The shortening of the latent period which occurred during the next 2 years seems to have taken place step-wise and not as a gradual decrease (Table I), and at the same time the susceptibility of the latent period to certain oestrogens was also reduced (Tables II and III). This increased independence of the hormonal environment with time conforms to the concept of progression (Foulds, 1949) and is paralleled by the behaviour of the interstitial cell tumour investigated by Gardner (1945) which eventually grew freely in both oestrogen and non-oestrogen treated animals. It is reasonable to suppose that the tumour used here was hormone-responsive when it arose, and if that were so the period of 10 years during which it remained responsive was a long one, being at least 4 times the normal life of a mouse.

The results of the experiments in Group B, although not directly comparable with those of Group A, are confirmatory in that the latent period of grafts was influenced by hormonal treatments in the same direction although not to the same extent.

As the latent period of grafts was less in castrated than in normal females, but was the same in both normal and castrated males, it follows that the delayed start of growth in intact females was due to inhibition by the ovarian secretion and not to a lack of stimulation by the testicular hormones.

Oestradiol dipropionate also considerably increased the latent period of the grafts and so it was presumably this component of the ovarian secretion which was the inhibiting agent. This effect of oestrogen is completely opposite to that observed by Gardner (1945) with a tumour of similar origin which grew only in the presence of oestrogen.

The latent period of 36 days which was observed in ovariectomised females (Experiment 4), where no oestrogen was present, was extended by 20 days when the observations were made in the presence of the ovarian secretion of normal females (Experiment 1). When, by injecting the hormone, the exposure to oestrogen was made continuous, the latent period (121 days) was 85 days longer. Thus an effective level of oestrogen could only have been present for a part of the time in the normal female.

The latent period of the grafts in normal females was not altered by testosterone injection (Experiment 5), although vaginal indications of oestrus were suppressed. This suggests that in the treated mice the level of oestrogen secretion was unaltered, and the castration effects of testosterone were not due to an inhibition of oestrogen secretion.

Oestradiol dipropionate and stilboestrol were equally effective in prolonging the latent period, whereas tri-phenyl-ethylene and the related compounds tri-phenyl-chlor-ethylene and $\alpha\alpha$-(p-hydroxyphenyl-$\beta$-bromo-ethylene) were inactive. As these five compounds all possess the usual properties associated with oestrogens, their capacity for tumour inhibition in these experiments must be a highly specific characteristic not implicit in oestrogenic activity.

It was found (Ellis, 1944) that some human mammary cancers, particularly among post-menopausal women, are inhibited or regress with oestrogen therapy. It may be, therefore, that some incipient malignant tumours of the breast are held in check by oestrogen during ovarian activity, but are able to grow without restraint when oestrogen secretion ceases at the menopause.

The latent periods of the auto-transplants (Table IV) were not significantly different from those of the initial grafts and it is interesting to note that a recurrence in the operation scar only arose after a similar lapse of time. Thus the considerable interval necessary before these grafts began to grow was consequent on the dislodging of the tumour cells from their established position and not to the strange environment of a new host.

Transplantation of a tumour, especially autotransplantation, may be regarded as experimental metastasis and Gardner (1945) has indicated the possible importance of hormonal mechanisms in the long delayed recurrences of primary tumours and growth of metastases in human cancer. It is obvious that if the latent period of metastases could be extended therapeutically beyond the normal life-span a virtual cure would be effected in those cases where the primary growth is removed.

### SUMMARY.

1. Eight to ten years after the first transplantation the latent period of a transplanted interstitial cell carcinoma of the testis, induced by triphenyl-ethylene in a Strong A mouse, was found to vary according to the hormonal environment of the host.

2. The latent period was similar in normal males or castrates of either sex, but was prolonged in normal females.

3. Oestradiol dipropionate and stilboestrol treatment of castrates extended the latent period beyond that observed in normal females, but three other oestrogens, tri-phenyl-ethylene, tri-phenyl-chlorethylene and $\alpha\alpha$-(p-hydroxyphenyl)-$\beta$-bromo-ethylene, had no effect. Testosterone treatment of normal females did not affect the latent period.

4. The tumour transplants became less susceptible to hormonal conditions during the 2 years of observation.

I am indebted to Dr. A. L. Walpole of the Imperial Chemical Industries Ltd., Manchester, who supplied the $\alpha\alpha$-(p-hydroxy-phenyl)-$\beta$-bromo-ethylene.

### REFERENCES.

Bonser, Georgiana, M.—(1942) *J. Path. Bact.*, **54**, 149.—(1944) *Ibid.*, **56**, 15.
*Idem* and Robson, J. M.—(1940) *Ibid.*, **51**, 9.
Ellis, F.—(1944) *Proc. Roy. Soc. Med.*, **37**, 731.
Foulds, L.—(1949) *Brit. J. Cancer*, **3**, 345.
Gardner, W. U.—(1943) *Cancer Res.*, **3**, 92.—(1945) *Ibid.*, **5**, 497.
Hooker, C. W., Gardner, W. U. and Pfeiffer, C. A.—(1940) *J. Amer. med. Ass.*, **115**, 443.
*Idem* and Pfeiffer, C. A.—(1942) *Cancer Res.*, **2**, 759.
Huseby, R. A.—(1954) *Proc. Amer. Ass. Cancer Res.*, **1** (No. 2), 21.
Shimkin, M. B., Grady, H. G. and Andervont, H. B.—(1941) *J. nat. Cancer Inst.*, **2**, 65.